# Evolution of Subclinical Hypothyroidism Diagnosed in the First 3 Months of Life in Newborns Living in North Italy: A Retrospective Cohort Study

**DOI:** 10.3390/children10010118

**Published:** 2023-01-06

**Authors:** Valentina Mancioppi, Valentina Antoniotti, Arianna Solito, Elisabetta Mingoia, Alice Monzani, Giulia Genoni, Ivana Rabbone, Flavia Prodam, Simonetta Bellone

**Affiliations:** 1 Division of Pediatrics, Department of Health Sciences, University of Piemonte Orientale, 28100 Novara, Italy; 2 Neonatal and Pediatric Intensive Care Unit, Maggiore della Carità University Hospital, 28100 Novara, Italy; 3 Endocrinology, Department of Translational Medicine, University of Piemonte Orientale, 28100 Novara, Italy; 4 Interdisciplinary Research Center of Autoimmune Diseases, University of Piemonte Orientale, 28100 Novara, Italy

**Keywords:** subclinical hypothyroidism, hyperthyrotropinemia, TSH levels, thyroid, therapy

## Abstract

Background: Subclinical hypothyroidism (SH) management in neonatal age opens important questions. We aimed to describe the evolution over time of subclinical hypothyroidism diagnosed in the first three months of life in a population of full-term neonates. Methods: A single-center longitudinal retrospective cohort study in a tertiary care center was conducted. We recruited 32 subjects with SH diagnosed within the first three months of life. We collected clinical, biochemical, and ultrasound data for every subject at the first examination and every six months until four years of age. Results: A total of 43.8% of subjects showed stimulating thyroid hormone (TSH) levels over the limit of 10 mUI/L and underwent treatment (Group 1). Eleven subjects started therapy at the first visit, while three subjects started it after a period of observation; 15.6% (Group 2A) showed a trend of TSH decrease and were finally discharged from the follow-up, while 40.6% (Group 2B) showed a TSH level slightly increased, changeless over time. Conclusions: We demonstrated that more than half of newborns with hyperthyrotropinemia did not require substitutive therapy showing a positive trend toward normalization or a remaining slight increase compared to normal levels. Moreover, our study suggests the need for a follow-up over time to check the TSH levels course.

## 1. Introduction

Subclinical hypothyroidism (SH) in neonatal age includes permanent and transient forms. Its management opens important questions in such a fragile period of life when thyroid hormones play a pivotal role in skeletal growth and neuro-cognitive development [1]. The prevalence of the different forms is presently unknown due to the presence of few studies with a small sample size of newborns [1,2]. Neonatal screening using TSH measurement has highly increased the detection of mild forms of SH, whose effect in later life is still a matter of debate.

Etiopathogenesis of SH may include physiological mechanisms of adaptation to new life and pathological maternal and perinatal conditions. Prematurity, being small for gestational age (SGA), genetic causes of dyshormonogenesis, or autoimmune diseases could differently affect thyroid function. Moreover, the environment could interfere with hormonal function, particularly iodine deficiency or exposure to disinfection agents rich in iodine [3,4].

The natural history of neonatal subclinical hypothyroidism is difficult to predict, ranging from normalizing TSH values to the evolution towards overt hypothyroidism, while often persistence of SH over time may exist. Several authors’ percentages of different clinical evolutions are variably reported [1,2,5,6,7].

Treatment of SH is still controversial, particularly in neonatal age. Levothyroxine (L-T4) treatment is recommended if TSH levels are persistent > 20 mIU/L, even with fT4 levels in the normal range. Conversely, if TSH levels fluctuate between 6 and 20 mUI/L, the European Society for Paediatric Endocrinology Consensus [3] suggests that both starting L-T4 therapy and a wait-and-see approach may be considered. Therefore, our study aimed to describe the characteristics and evolution over time of subclinical hypothyroidism diagnosed in the first three months of life in a population of full-term neonates of several ethnic origins living in North Italy.

## 2. Materials and Methods

### 2.1. Study Design and Population

We conducted a single-center retrospective cohort study. We included all the subjects with neonatal SH diagnosed into the first three months of life referring to the Paediatric Endocrinology Unit of the University Hospital ‘Maggiore della Carità of Novara, in Northern Italy, between October 2012 and June 2016. In our country, all neonates undergo a neonatal screening between 48 and 72 h from birth. If TSH determination is >8 mU/L at the first screening, a second confirmatory screening is performed. If TSH is >6.5 mU/L, the patients must be evaluated by a pediatric endocrinologist, and serum fT4 and TSH are measured. If TSH is >16 mU/L at the first screening, patients must go directly to a pediatric endocrinologist, and serum fT4 and TSH is measured. In the neonatal period, SH was biochemically defined as a serum TSH concentration between 6 and 20 μIU/mL in the presence of serum-free T4 (fT4) concentration within its normal reference range (0.89–1.76 ng/dL). For subjects that were more than one month old, SH was defined by a TSH value between 4.5 and 10 μIU/mL. Exclusion criteria were overt congenital hypothyroidism, patients with chronic disease, syndromes, born premature or SGA, being on chronic therapy, or twins. All patients resided in an area by the Mediterranean Sea in an iodine-sufficient population.

For every subject, we collected clinical, biochemical, and ultrasound data at the time of the first medical examination and about every six months until four years of age. We retrospectively divided our patients into two groups based on the necessity to start therapy with L-thyroxine during the time of observation (4 years): Group 1 consisted of treated children, and Group 2 consisted of untreated children whose TSH levels normalized during the follow-up or remained slight increase compared to normal levels. Our patients were therefore evaluated by comparing these two groups.

### 2.2. Clinical and Auxological Data

Anamnestic data were collected: first and second-degree positive family history of thyroid diseases, maternal thyroid disease and treatment during pregnancy, and presence of gestational diabetes.

Clinical and auxological characteristics were recorded for each patient at every medical examination. The length, weight, and cranial circumference measured at birth were classified as Adequate for Gestational Age (AGA) according to Italian growth charts [8]. The length was measured by a standard infant stadiometer to the nearest 0.5 cm until 24 months of age and expressed according to WHO growth charts for children within two years of age [9]. From 24 months of age, height was measured by a Harpenden stadiometer to the nearest 0.5 cm and expressed according to the Italian cross-sectional 2006 growth charts [10]. Length and height measures were repeated three times, and the mean values were considered. Parental target height and its range (TH ± 8.5 cm) were calculated. Weight was measured by manually operated balance to the nearest 100 g and expressed according to WHO growth charts [9]. Cranial circumference was measured using a measuring tape that cannot be stretched, wrapping the tape around the widest possible circumference of the head to the nearest 0.1 cm according to WHO growth charts for children within two years of age [9].

### 2.3. Biochemical Parameters and Imaging Analysis

TSH, fT4, fT3, anti-thyroperossidasis (TPOAb), and anti-thyroglobulin antibodies (TGAb) were measured by highly chemiluminescent immunoassays technology with acridinium ester as a label and paramagnetic particles as a solid phase (Advia Centaur Immunoassy System; Siemens Heathcare Diagnostics, Deerfield, IL, USA). Sensitivity was 0.080 mUI/L for TSH, 0.2 pg/mL for FT3, 0.1 ng/dL for FT4, 30 UI/mL for TGAb, and 25 UI/mL for TPOAb assay. Reference values were 0.450–3.500 μIU/mL for TSH, 2.30–4.20 pg/mL for fT3, and 0.89–1.76 ng/dL for fT4.

Thyroid morphology was evaluated by ultrasound, and anteroposterior and transverse diameters were measured using a high-resolution 7–13 MHz linear transducer, positioning the newborn with his neck slightly extended. Thyroid tissue echogenicity was evaluated in a longitudinal scan of the thyroid lobes by a standardized comparison with the echogenicity of the adjacent muscles and categorized as normal, decreased, or increased compared to them. An ultrasonographic pattern of thyroiditis was considered as the presence of multiple hypoechoic foci or patches scattered throughout an otherwise normal echogenic gland or a gland with diffuse hypoechogenicity compared to the anterior strap muscle.

### 2.4. Population Characteristics

We enrolled 32 subjects with SH diagnosed within the first three months of life (mean age 43 ± 4.8 days). Subjects were referred to our Pediatric Endocrinology Unit immediately after birth or within 3 months for a clinical picture suggestive of thyroid disease in the Neonatal Unit: 24 subjects (75%) due to a recall after pathologic neonatal screening, 4 subjects (12.5%) for familiarity (mother with autoimmune thyroiditis or siblings with known thyroiditis from birth), and 4 subject (12.5%) for neonatal jaundice or failure to thrive. Eighteen were Females (56.2%), and fourteen were males (43.8%); most of the patients were of Caucasian origin (91%), while 9% were of North African origin. A positive family history of thyroid diseases was present in 53.1% of subjects (60% of maternal origin). Four mothers (12.5%) reported hypothyroidism onset during pregnancy, well controlled with L-thyroxine treatment. Most newborns received breastfeeding (64.1%), 20.5% formula feeding, and 15.4% mixed feeding.

### 2.5. Statistical Analysis

Samples of 23 individuals were estimated enough to demonstrate a difference of 2.8 mcUI/mL of TSH to reach the upper limit of our laboratory range with a significance level of 95% based on previous TSH data with an SD of 2.3 [7,11]. Furthermore, a reduction of 50% in TSH data needs 17 individuals based on these data [7,11]. Data were expressed as mean ± mean standard error (SEM), percentages, or absolute numbers, as appropriate. Continuous data were compared by nonparametric tests, such as Mann–Whitney U test or Wilcoxon test, where appropriate. Dichotomous and categorical data were compared using the chi-square test. Measures repeated in time were analyzed using ANOVA for repeated measures with timing as an intra-subject variable and treatment as an inter-subject variable. Correlations were examined using Pearson correlation coefficients ^®^. Skewed variables were log-transformed before the analysis. Statistical significance was assumed at *p* < 0.05. All analyses were performed using SPSS (version 26.0, IBM Corp, Armonk, NY, USA).

## 3. Results

### Clinical, Biochemical, and Instrumental Data at First Visit and during Follow-Up

The mean TSH value at the first visit was 15.1 ± 2.8 mcU/mL, and FT4 and FT3 levels were always in the normal range (mean ± SEM 1.4 ± 0.1 and 4.9 ± 0.1 ng/dL, respectively), accordingly to our inclusion criteria. At least one of TPOAb and TGAb was positive in nine subjects (28.2%). Thirty newborns showed normal size and echographic pattern (93.8%), one patient had a hypoplasic gland (3.1%), and one other diffuse hypoechogenicity (3.1%).

At baseline (43 days from the birth’s day), TSH values were negatively correlated with weight (R = −0.354, *p* < 0.02) and cranial circumference (R = −0.394, *p* < 0.028) and positively correlated with paternal age (R = 0.296, *p* < 0.05), family history for thyroid diseases in first and second-grade relatives (R = 0.412, *p* < 0.03), gestational diabetes (R = 0.258, *p* < 0.05), and nearly to significance with maternal age (R = 0.205, *p* < 0.07).

Auxological data are reported in Table 1.

Among Group 1, which consisted of treated children, 14 subjects (43.8%) globally showed TSH levels over the limit of 10 mUI/L and underwent treatment, according to international guidelines in neonatal age [3] and parental agreement. Eleven subjects started therapy at the first visit, while three subjects started it after a period of observation (between 6 and 12 months). Regarding these three patients, their TSH levels progressively increased from a mean TSH baseline level of 6 up to reach a value > 10 UI/L, which corresponded with the treatment’s start. Starting therapy of L-thyroxine at the first visit was at a mean of 28 + 2.8 days. Starting mean dose of L-thyroxine was 4.5 ± 0.4 mcg/kg/die. Drug formulation was a liquid formulation (drops) for all neonates. Group 2 was characterized by patients who showed a trend of TSH decrease and did not need to start treatment with L-thyroxine. Looking more specifically at this group, it consisted of children who were finally discharged from the follow-up (five subjects, 15.6%) (Group 2A) and patients who showed a TSH level slightly increased, changeless over time (13 subjects, 40.6%) (Group 2B). TSH levels at baseline were significantly higher in the group of neonates who started L-T4 therapy compared to not treated group (13.1 ± 2.1 vs. 8.2 ± 1.8 mcU/mL; *p* < 0.001). In the subsequent visits, the subject’s number was progressively reduced, due to TSH normalization among patients of Group 2 who were referred to a general pediatric practitioner for clinical evaluations, except for one patient who moved to another city. Treated patients (Group 1) normalized their TSH value within 2 weeks. In Group 2A, TSH levels normalized within 18 months (6 ± 6, mean and SD). In Group 2B, TSH levels always remained above the normal range.

**Figure 1:**
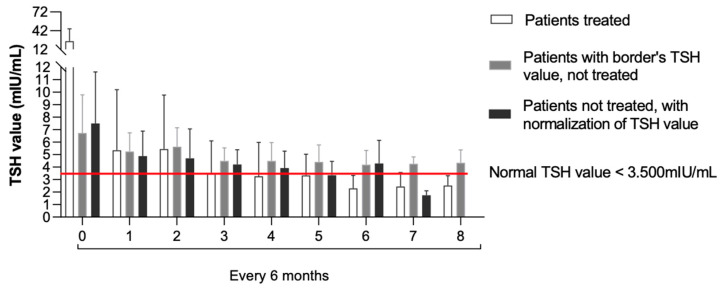
TSH levels’ trend evaluated every six months until 4 years of age among the three groups.

Figure 1 describes the TSH levels’ trend among the groups at the assessments carried out every six months until four years of age.

Supplementary Table S1 shows the thyroid function of Group 1 and Group 2 during the follow-up.

**Figure 2:**
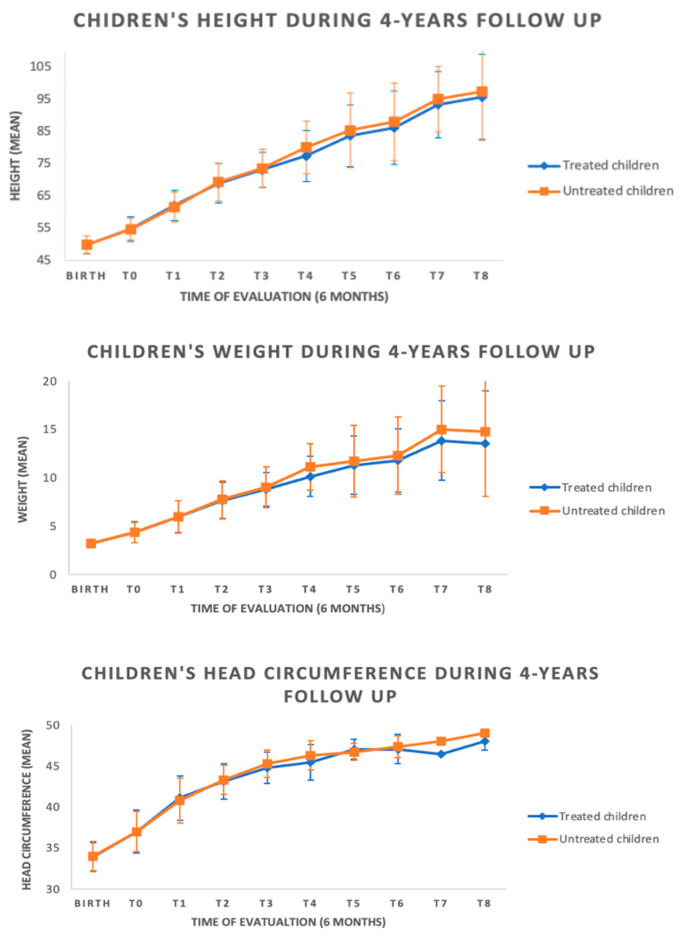
Auxological data during follow-up among the groups.

All subjects showed normal growth for all parameters (length, weight, and cranial circumference) over time (see Figure 2), without differences among the two groups.

Globally, only nine patients (21.8%) showed positivity of antithyroid antibodies at first evaluation; three (6.2%) became autoantibodies positive between 6 and 12 months. Among these patients, one subject (33%) has started therapy with L-thyroxine (Group 1), while the others belonged to the not-treated groups (Group 2). After the positivity, patients underwent a thyroid ultrasound, which was normal. By contrast, six patients (16%) demonstrated a change from positivity to normalization, with patients divided equally between the treated and untreated groups. The antibodies normalized on average within ten months.

All patients underwent a thyroid ultrasound, and 93.8% were normal; in one case (3.1%), the presence of thyroid hypoplasia was described, and the echography pattern was compatible with thyroiditis in one patient.

## 4. Discussion

Our study aimed to describe neonatal SH’scharacteristics and progression over time. The scientific literature about this topic in neonatal age is discordant and inconclusive. While congenital permanent forms are mainly known, subclinical hypothyroidism might be caused by milder forms of thyroid dysfunction, including genetic or environmental causes [1,3,4].

The apparent incidence of this pathology is changed primarily due to the increased stringency of newborn screening [12] and the use of more sensitive TSH arrays, leading to increased detection of milder cases [6]. Another cause may be the increased percentage of high-risk conditions as preterm newborns, twins, and SGA neonates, related to improved skills in neonatal resuscitation and requiring repeated screening tests as suggested by international guidelines [3,13].

The management of SH in neonatal age is still a matter of debate, and different clinical approaches are performed by clinicians [4,14].

We evaluated the clinical and hormonal course every six months until four years of age. Due to the study’s retrospective nature, patients who normalized their thyroid function withdrew from the follow-up; consequently, the number of subjects decreased over time from 32 to 19. One child was lost at follow-up because of a transfer to another city. About forty percent of the patients had transient hypothyroidism, reaching euthyroidism within three years of age, with most of them within six months. Only 15.6% of the subjects maintained a condition of slight hyperthyrotropinemia, while 43.8% of patients were treated with replacement therapy. Therefore, our study demonstrated that 56.2% of newborns did not require substitutive therapy showing that the natural history of this condition comprises a positive trend toward normalization or a permanent slight increase compared to normal levels.

Data in the literature about the natural course of this condition are controversial; several studies tried to assess the predictive factors of evolution to persistent or transient SH, despite the complexity of recognizing them. As in our study, Leonardi et al. [11] demonstrated that 36% of neonates with subclinical hypothyroidism normalized TSH levels in the first years of life, while Aguiar et al. [4] showed a transient SH in 46% of newborns. Highest TSH values on neonatal screening were often found in preterm or small for gestational age newborns [2,15], while Unuvar et al. [14] showed no differences. Data in our population are similar to these findings, but we must consider that our population includes only at-term newborns, with few risk factors in this point of view.

We showed that subclinical hypothyroidism was more common in infants with a positive family history of thyroid dysfunctions, mainly in the presence of a familiar dysthyroidism in the mother’s family (60% of our patients). Other authors, such as Aguiar et al. [4], proved a major risk of hyperthyreotropinemia in infants born to mothers suffering from hypothyroidism or Graves’ Disease caused by transplacental transfer of maternal antibodies. Idris et al. [16] highlighted the importance of ensuring a normal maternal thyroid function during pregnancy. In fact, thyroid hormones exert their crucial role during early brain development through the regulation of gene expression, involving epigenetic mechanisms in fetal programming via maternal thyroid dysfunction. At present, the real impact on infant neurodevelopment is not yet fully understood, and maternal euthyroidism (TSH values in range according to gestational age) is still a topic of prolonged debate in the endocrinological community.

The most common cause of hyperthyrotropinemia is an increase in thyroid antibodies [4,7]. In our population, only 21.8% showed positivity of antithyroid antibodies at first evaluation, but 6.2% showed a change in becoming autoantibodies positive over time. By contrast, 16% demonstrate a change from positivity to normalization. We supposed that infants with transient serum autoantibodies have a major risk of thyroid dysfunction due to maternal autoimmune disease via trans-placental autoantibodies transfer during pregnancy. Otherwise, patients with previously negative serum antibodies, becoming positive hereafter, should demonstrate latent thyroid dysfunction and more probably need replacement therapy. Zung et al. [15] suggested the importance of evaluating thyroid ultrasonographic characteristics to predict the natural course in these patients: the presence of abnormal thyroid imaging would be predictive of persistent hyperthyreotropinemia. Instead, in our population, US characteristics at baseline resulted almost completely normal, except for one case of thyroid hypoplasia and one patient whose echography pattern was compatible with thyroiditis.

Whether this condition needs to be treated is still a matter of debate. Some authors suggested that subjects with mild hyperthyreotropinemia should be tracked without replacement therapy, monitoring in the meantime thyroid function values and auxological parameters [5,11]. In case of persistent hyperthyreotropinemia, patients should be treated with Levothyroxine (L-T4) to normalize their TSH value permanently. By contrast, Daliva et al. suggested replacement therapy in all neonates with hyperthyreotropinemia, either in case of severe TSH elevation or in borderline TSH [17].

Since no definitive data are available on the long-term neurological effects of SH in neonatal age [3], according to ESPE guidelines, we decided to treat infants who showed persistent TSH levels of more than 10 mUI/L, discussing with the family this opportunity.

It has been demonstrated that newborns with transient neonatal hyperthyrotropinemia present a higher risk of subclinical hypothyroidism in infancy and early childhood [5]. Several factors have been proposed as biomarkers of persistency as male gender, greater maternal age and perinatal stress, thyroid imaging [7], or a high L-thyroxine (L-T4) dose required to maintain a euthyroid state [14]. Our group’s median dose of L-thyroxine was 4.34 mcg/kg at baseline, slightly higher than other authors’ reports [14].

Our study had some limitations. First, our study was retrospective, and some data could be missed. Moreover, our sample size is small, and due to the study’s design, it further decreased over time. The strength of our study is related to the limited number of studies found in the literature regarding subclinical hypothyroidism in newborns. The treatment of SH is still controversial, particularly in neonatal age, and this topic is still debated among both pediatric and adult endocrinologists. A further important aspect is the relatively long follow-up (4 years) during which our patients were monitored both clinically and biochemically to assess the evolution over time of subclinical hypothyroidism.

## 5. Conclusions

In conclusion, our longitudinal retrospective study demonstrated that more than half of newborns with hyperthyrotropinemia did not require substitutive therapy showing a positive trend toward normalization or a remaining slight increase compared to normal levels. Moreover, our study suggests the need for a follow-up over time to check the TSH levels course. More extensive studies until adulthood are necessary to establish criteria that might predict the normalization or deterioration of hormonal patterns. Whether this condition needs to be treated remains still matter of debate. Furthermore, no definitive data are available on the long-term neurological effects of SH in neonatal age, but neither about the benefits of treatment on neurocognitive development and growth. Since the first year of life is a delicate period for neurological development and growth, peculiar attention could be used by clinicians involving families in discussing different opportunities.

## Figures and Tables

**Table 1:** Clinical characteristics of treated (Group 1) and untreated (Group 2) patients.

	Group 1	Group 2
Number	14 (44%)	18 (56%)
Female	7 (50%)	10 (55%)
Caucasian ethnicity	14 (50%)	15 (83%)
Gestational Age (weeks)	39 ± 0.10	39 ± 0.06
Mother’s thyreopathy	0 (0%)	4 (22%)
Levothyroxine during pregnancy	0 (0%)	3 (17%)
Age at first visit (days)	42.5 ± 0.8	42.2 ± 0.8
Length at first visit (SDS)	1.08	1.09
Weight at first visit (SDS)	0.70	0.69
HC at first visit (SDS)	1.23	1.32

All data are expressed as mean ± sem (standard error of the mean) or percentage. Abbreviations: HC: head circumference; SDS: standard deviation scores.

## Data Availability

The datasets for this article are not publicly available due to concerns regarding participant/patient anonymity. Requests to access the datasets should be directed to the corresponding author.

## Supplementary Materials

The following supporting information can be downloaded at: https://www.mdpi.com/article/10.3390/children10010118/s1, Table S1: Thyroid function of treated (Group 1) and untreated (Group 2) patients during the follow up.
