# Supplementary material for: Evolution of Subclinical Hypothyroidism Diagnosed in the First 3 Months of Life in Newborns Living in North Italy: A Retrospective Cohort Study
Source: Children (Basel). 2023 Jan 6;10(1):118. doi: 10.3390/children10010118 (PMC9856945; doi:10.3390/children10010118)
Supplement: Supplementary file 1 [file children-10-00118-s001.zip › children-2134044-supplementary.pdf]

Supplementary Table 1. *Thyroid function of treated (Group 1) and untreated (Group 2) patients during the follow up*

	TSH (mIU/ml)		fT3 (pg/ml)		fT4 (ng/dl)	
	**Group 1**	**Group 2**	**Group 1**	**Group 2**	**Group 1**	**Group 2**
**T0**	15.74±2.09	7.02±5.05	4.92±0.35	4.90±0.36	1.44±0.19	1.42±0.22
**T1**	5.33±3.06	5.19±1.50	4.80±0.26	4.76±0.25	1.53± 0.24	1.46±0.29
**T2**	5.34±1.72	5.10±1.15	4.68±0.28	4.53±0.30	1.49±0.29	1.44±0.35
**T3**	3.46±1.05	4.20±0.87	4.26±0.43	4.24±0.40	1.56±0.53	1.52±0.52
**T4**	3.09±0.79	4.10±0.76	4.30±0.25	4.30±0.23	1.63±0.56	1.58±0.59
**T5**	3.09±0.87	3.87±0.70	4.28±0.39	4.27±0.35	1.41±0.12	1.36±0.15
**T6**	2.54±0.92	4.15±0.76	4.34±0.30	4.24±0.36	1.38±0.11	1.35±0.14
**T7**	2.59±0.68	3.15±1.07	4.20±0.24	4.18±0.27	1.39±0.11	1.39±0.11
**T8**	2.69±1.05	4.16±0.68	4.28±0.27	4.29±0.25	1.42±0.15	1.40±0.16

Thyroid function assessed at the time of the first medical examination (T0) and every six months until four years of age (T1-T8).
All data are expressed as mean ± sem (Standard Error of the Mean).
Abbreviations: fT3, free tri-iodothyronine; fT4, free thyroxine; TSH, thyroid stimulating hormone.
